# Comparison of Ventilator-Associated Pneumonia in Patients Admitted to Intensive Care for COVID-19 Versus Other Reasons: A Single-Centered Study

**DOI:** 10.5152/TJAR.2022.21310

**Published:** 2022-04-01

**Authors:** Ahmet Sarı, Hilal Akça, Damla Akman, Asuman İnan, Aytekin Kaymakçı, Osman Ekinci

**Affiliations:** 1 Department of Anaesthesiology and Reanimation ICU, HSU Haydarpaşa Numune Training and Research Hospital, İstanbul, Turkey; 2 Department of Infectious Diseases and Clinical Microbiology, HSU Haydarpaşa Numune Training and Research Hospital, İstanbul, Turkey; 3 HSU Haydarpaşa Numune Training and Research Hospital Management, İstanbul, Turkey

**Keywords:** Coronavirus, COVID-19, secondary infection, Microorganisms, SARS-CoV-2, ventilator-associated pneumonia

## Abstract

**Objective:**

COVID-19 patients in intensive care usually need invasive mechanical ventilation due to advanced respiratory failure. Deep lymphopenia, immunosuppressive agents, long-term mechanical ventilation, and sedation may lead to ventilator-associated pneumonia; an important cause of morbidity and mortality. This study evaluates the frequency, clinical features, causative pathogens, and outcomes of ventilator-associated pneumonia in COVID-19 patients who require mechanical ventilation.

**Methods:**

The files of patients hospitalized in our hospital’s intensive care clinic between March 25, 2020, and January 15, 2021, in the first 2 peaks due to COVID-19 and other reasons were retrospectively reviewed.

**Results:**

We found ventilator-associated pneumonia rate in COVID-19 patients as 52.2%, which was statistically significantly higher than in non-COVID patients (33.5%). Purulent sputum, leukocyte, and procalcitonin levels were found to be significantly higher in both groups developing ventilator-associated pneumonia. However, fever levels were found to be significantly normal in both groups; 97.1% and 87%, respectively. High fever was observed in only 2.9% of COVID-19 patients who developed ventilator-associated pneumonia. We determined a mortality rate of 17 (100%) in the diabetes patients in the COVID-19 group, which was statistically significantly higher than in non-COVID-19 patients at 9 (64.3%). The mortality rate (86.1%) in those with COVID-19 was statistically significantly higher than in those without COVID-19 (64.9%).

**Conclusions:**

Ventilator-associated pneumonia is more common in COVID-19 patients treated with mechanical ventilation than in non-COVID patients. The predictive value of fever in the diagnosis is very low, and agent production together with increased purulent sputum will be more valuable in terms of diagnosis.

**Main Points**

- The incidence of Ventilator-Associated Pneumonia (VAP) in Covid-19 patients was found to be higher, 52.2% to 33.5%.
- Mortality is higher in Ventilator-Associated Pneumonia (VAP) cases developing in Covid-19 patients (86.1%-64.9%), and this rate is much higher in diabetic Covid-19 patients (100%-64.3%).
- The predictive value of fever in the diagnosis of VAP is low in both groups.

## Introduction

COVID-19 disease is a clinical situation that is caused by severe acute respiratory syndrome coronavirus-2, which usually progresses with mild and moderate clinical findings, but a small percentage of the cases manifest in the form of severe pneumonia cases which require intensive care treatment.^1,2^ Pneumonia that is observed in these cases may present with conditions such as fever, lymphopenia, leukocytosis, elevated D-dimer and ferritin levels, hypoxemia, bilateral infiltrates, and even multiple organ failure.^3,4^

Mechanical ventilation (MV) has an important place in the treatment of COVID-19 patients in intensive care. Prolonged hospitalization in MV intensive care units (ICUs) can cause ventilator-associated pneumonia (VAP), which is an important cause of morbidity and mortality, as well as increased costs. In COVID-19 patients, reasons such as deep lymphopenia and virus-related immunosuppressive effect, immunosuppressive conditions caused by steroids and similar immunosuppressive agents, and long-term sedation cause VAP to develop more easily when MV is administered.^5^ Ventilator-associated pneumonia is defined as a pulmonary parenchymal infection that develops in patients who receive MV for at least 48 hours.^6^

Ventilator-associated pneumonia is frequently investigated among COVID-19 patients. According to different studies, its incidence ranges from 36% to 85%, and mortality rates in ICUs range from 29% to 43%.^5,7-9^ In addition, VAP in COVID-19 is associated with increased mortality at 28 days.^10^ The characteristics and specific risk factors of VAP in COVID-19 patients have not been determined yet.^11^

The aim of this study is to investigate the differences in VAPs in terms of clinical, microbiological, and biochemical properties in COVID-19 patients and non-COVID patients. The main hypothesis that needs to be validated is that VAP in COVID-19 is a new “pathology” with some characteristics which require different healthcare services than VAP in non-COVID patients.^12^

## Methods

After obtaining institutional and local ethics committee permissions (HNEAH-KAEK 2020/70) for our study, the files of patients hospitalized in the intensive care clinic of our hospital between March 25, 2020, and January 15, 2021, in the first and second peaks due to COVID-19 and non-COVID reasons were retrospectively reviewed. Patients who developed VAP in both groups were recorded. Ventilator-associated pneumonia diagnosis was established based on the increase in fraction of inspired oxygen (FiO_2_) and positive end expiratory pressure (PEEP) levels which occurred 48 hours after invasive ventilation treatment, the existence of parameters, that is, fever (>38°C or <36°C), leukocytes (<4000 or >12 000 at mm^3^) which support infection with continued use of one or more antibiotics for at least 4 days, increased pulmonary secretions and existence of growth in tracheal aspirates taken, and/or observation of ≥25 neutrophils and ≤10 epithelial cells in all fields in aspirate/bronchoalveolar lavage (BAL) sample.^13^ In order to prevent VAP development in both groups in intensive care; practices such as hand washing, raising the head of the bed, oral care with chlorhexidine, daily sedation holidays were attempted per the requirements of the patients’ clinics.

In our study, the VAP rates, comorbidities, types of antibiotics used, the relationship between comorbidities and mortality, the microorganisms that cause VAP, and the relationship between VAP rates and mortality were evaluated in COVID-19 and non-COVID patients.

The main outcome was VAP incidence and mortality rates in the intensive care unit. Secondary outcomes were the determination of agents causing VAP, their relationship with comorbidities, and the antibiotics used.

### Statistical Analysis

To evaluate the findings obtained in the study, the Statistical Package for Social Sciences version 22.0 software (IBM Corp.; Armonk, NY, USA) was used for statistical analysis. While evaluating the study data, the appropriateness of the parameters to normal distribution was evaluated using the Shapiro–Wilks test. Descriptive statistical methods (mean, standard deviation, and frequency) were used while evaluating the study data. In addition to these, Mann–Whitney *U* test was used for comparisons between 2 groups that did not exhibit a normal distribution. Chi-square test, Fisher’s exact test, and Continuity (Yates) Correction were used to compare qualitative data. Spearman’s rho correlation analysis was used to examine the relationships between parameters that did not conform to the normal distribution. Significance was evaluated at the *P *< .05 level.

## Results

Our study was conducted between March 25, 2020, and January 15, 2021, with 126 cases aged between 21 and 99 years. Eighty-two (65.1%) of the cases were male and 44 (34.9%) were female. The mean age of the cases was 71.21 ± 13.67 years and the median was 74 years. The study was examined under 2 groups, namely 54 (42.9%) non-COVID and 72 (57.1%) COVID-19 patients.

Ventilator-associated pneumonia rate in the COVID-19 group (52.2%) was found to be statistically significantly higher than in non-COVID patients (33.5%) (*P = *.001; *P *< .05).

There was no statistically significant difference between the COVID-19 and non-COVID groups in terms of age and gender (*P *> .05) (Table 1).

While the body temperature was normal in 92.7% of the cases, it was high in 7.3%. While the leukocyte level of 82.5% of the patients was abnormal, 17.5% of them were normal. While 29.8% of the patients had normal procalcitonin levels, 70.2% of them had high procalcitonin levels. Purulent sputum was not observed in 5% of the patients, while it was observed in 95%. A total of 74.4% of the patients were sedated. While 76.9% of the cases died at the intensive care unit, 0.79% were hospitalized and 22.2% were discharged (Table 2).

The duration of treatment, MV duration, and ICU duration of COVID-19 patients were found to be statistically significantly lower than non-COVID patients, and the rate of sedation administering was higher (*P  = *.010; *P *< .05). The incidence of high fever (13%) in non-COVID patients was found to be statistically significantly higher than in COVID-19 patients (2.9%) (*P = *.037; *P *< .05) (Table 3).

Mortality was found to be statistically significantly higher in COVID-19 patients (86.1%) than in non-COVID (64.9%) patients (*P  = *.012; *P *< .05) (Table 3).

Purulent sputum, leukocyte, and procalcitonin levels were found to be significantly higher in both groups developing VAP. The body temperature level was found to be normal in the COVID-19 and non-COVID groups, at 97.1% and 87%, respectively. High body temperature was detected in only 2.9% of COVID-19 patients who developed VAP, while high temperature was detected in 13% of non-COVID patients (Table 3).

There was no statistically significant difference in comorbidity rates between COVID-19 patients and non-COVID patients (*P *> .05) (Table 4).

There is no statistically significant difference in the incidence of congestive heart failure, chronic obstructive pulmonary disease (COPD), coronary artery disease (CAD), hypertension (HT), diabetes mellitus (DM), dementia, chronic renal insufficiency, malignancy, atrial fibrillation (AF), Parkinson’s, cerebrovascular event, and asthma comorbidities between COVID-19 and non-COVID patients (*P *> .05) (Table 4).

When the effects of commonly encountered comorbidities on mortality were compared, no difference was found between the groups in terms of COPD, CAD, HT, and malignancy. The mortality rate in the COVID-19 group in DM patients was 17 (100%), which was statistically significantly higher than 9 (64.3%) non-COVID patients (*P  = *.012; *P *< .05).

There is no statistically significant difference between COVID-19 and non-COVID patients in terms of reproduction rates caused by *Stenotrophomonas*, *Acinetobacter*, *Staphylococcus* *aureus*, *Pseudomonas aeruginosa*, and *Klebsiella pneumoniae* (*P *> .05).

Antibiotics were used in all cases. Among the cases, 34.1% used colimycin, 7.9% used tigecycline, 58.7% used meropenem, 27% used teicoplanin, 0.8% used azithromycin, 3.2% used ceftriaxone, 50% used piperacillin-tazobactam, 3.2% used daptomycin, 1.6% used linezolid, 4.8% used trimethoprim+sulfamethoxazole, 0.8% used moxifloxacin, 4% used cefepime and 1.6% used antifungal (Table 5).

The rate of using teicoplanin in COVID-19 patients (38.9%) was found to be statistically significantly higher than non-COVID patients (11.1%) (*P  = *.001; *P *< .05).

There is no statistically significant difference in the rates of use of commonly used antibiotics colimycin, tigecycline, meropenem, piperacillin-tazobactam, and trimethoprim + sulfamethoxazole between COVID-19 and non-COVID patients (*P *> .05) (Table 5).

Multi-drug-resistant microorganisms were observed in 46 (63.8%) COVID-19 patients, while this rate was found to be 37 (68.5%) in the non-COVID group, and there was no significant difference between the groups in terms of resistance status. (*P *> .05) (Table 5).

## Discussion

One of the most important problems we encounter in invasive MV practices in COVID-19 patients is VAP. In COVID-19 patients, increased susceptibility to bacterial superinfection, in addition to lung injury caused by COVID-19, virus-induced immunosuppressive effect with profound lymphopenia, anti-inflammatory or immunosuppressive situations caused by steroids, etc. (i.e., anti-IL-6 receptor monoclonal antibodies), anti-inflammatory or immunosuppressive cases caused by immunosuppressive agents, increased workloads due to the pandemic, reduction in meticulous utilization of standard prevention strategies and extended use of sedation^14-17^ can play roles in the development of VAP. Due to severe clinical status in COVID-19 patients, long-term sedation and MV cause further VAP development. Ventilator-associated pneumonia is frequently observed in ICUs, especially in patients receiving prolonged MV support, and it causes increases in both cost and mortality.^18^ In their study with the participation of 586 COVID-19 patients, Giacobbe et al^10^ found that VAP developed in 171 (29%) patients, and *P. aeruginosa* and *S. aureus* growth was the most common in these patients. In their study, Karakuzu et al^19^ found *Acinetobacter baumannii*, *P. aeruginosa*, and methicillin-resistant *S. aureus* as the most common causative microorganisms for VAP. In their study, Blonz et al^16^ reported that the most frequently encountered VAP factor in COVID-19 patients were Enterobacteria, which comprise 49.8% of all isolated pathogens. In their study, Ippolito et al^9^ demonstrated that the risk of developing VAP in patients with COVID-19 may be higher than in patients without COVID-19. They found the VAP development rate to be 45.4% and the mortality rate to be 42.7%.^9^ In our study, the incidence of VAP in cases with COVID-19 (52.2%) was found to be statistically significantly higher than in non-COVID patients (33.5%). There was no difference between the agents that are isolated in tracheal aspirate samples in both groups, and the most common agents were determined as *A. baumannii*, *P. aeruginosa*, and *K. pneumoniae*.

Patients with severe COVID-19 usually carry the main risk factors (hypertension, chronic obstructive pulmonary disease, chronic renal failure, length of stay in ICU, multiple organ failure, and low blood oxygen level) observed in VAP caused by *A. baumannii*.^20^ *A. baumannii* is widely resistant to disinfection, a polysaccharide capsule and the formation of biofilms contribute to the high pathogenicity of this bacterium.^21^ Rezai et al^22^ found in their study that *A. baumannii* was responsible for approximately 47% of VAP cases in ICUs. In our study, the rate of VAP in COVID-19 patients (52.2%) and which was approximately 1.5 times that of non-COVID cases, while mortality values were determined likewise as 86.1% and 64.9%, respectively. The most reproducing agent in COVID-19 and non-COVID cases was *A. baumannii*, which reproduced by 40 (55.6%) and 35 (64.8%), respectively, and mortality in these patients was 34 (85%) and 21 (60%), respectively, thus higher in COVID-19 patients. In line with these findings, we can say that VAP caused by *A. baumannii* increases mortality more in COVID-19 patients. Measures such as strict adherence to infection control measures and use of VAP bundles can prevent the development of such cases and reduce mortality.^23,24^

The clinical features of COVID-19 pneumonia and VAP are similar and usually present as high fever, severe hypoxemia, leukocytosis, biological inflammatory syndrome, and extensive bilateral radiological changes.^25^ One of the parameters used in diagnosing VAP is body temperature being >38°C or <36°C, which is accepted as a positive finding.^13,26^ In our study, body temperature was found to be normal in 87% and high in 13% of non-COVID cases, and normal in 97.1% and high in only 2.9% of COVID-19 cases. Therefore, we believe that the predictive value of body temperature in diagnosing VAP is low. On the other hand, the presence of procalcitonin, leukocyte elevation, and increased purulent sputum was found to be significantly higher in both groups, and we believe that these parameters will be more valuable in diagnosing VAP, especially with a positive culture. In their study, Hodges et al^27^ found high procalcitonin levels in COVID-19 patients hospitalized in the intensive care unit and associated it with high mortality. Côrtes et al^25^ evaluated procalcitonin as a biomarker for the diagnosis of VAP in their study in patients with COVID-19 and found that high procalcitonin levels were valuable in terms of diagnosis, in line with our results. In our study, the use rate of teicoplanin in COVID-19 patients in the treatment of VAP was statistically significantly higher than those with non-COVID patients, and it was observed that it was similar in both groups in terms of other antibiotics, especially colimycin, tigecycline, meropenem, piperacillin-tazobactam.

Treatment duration, MV duration, and ICU duration in COVID-19 patients were found to be statistically significantly lower than in non-COVID patients. We believe that this result is rather related to the early death of our COVID-19 patients.

Type 2 diabetes is regarded as a chronic and low-grade chronic inflammatory disease caused by long-term immune system imbalance, metabolic syndrome, or obesity associated with excessive food intake.^28,29^ In their study, Guo et al^30^ found that Type 2 Diabetes, which is currently a common disease, is associated with poor prognosis and higher mortality in COVID-19 patients. In our study, the mortality rate in the COVID-19 group in DM patients was 17 (100%), hence statistically significantly higher than the rate of 9 (64.3%) in non-COVID patients. DM is an important comorbidity that increases mortality in COVID-19 patients. For this reason, we should use these drugs carefully, keeping in mind that the steroids we use in the treatment of COVID-19 patients with diabetes may have negative effects on the regulation of diabetes.

In their study, Yang et al^31^ found the growth rate of tracheal aspirate samples taken after intubation to be 58.3% in COVID-19 patients. In our study, the VAP rate in the COVID-19 group (52.2%) was found to be statistically significantly higher than in non-COVID patients (33.5%). There are studies showing that deep lymphopenia and virus-related immunosuppressive effect are the main reasons for the increased incidence of infections in COVID-19 patients, as well as steroids used to control the hyperinflammatory response that emerges.^5,32^ In our study, while steroids were used in 43 (59.7%) patients in the COVID-19 group where more infections were encountered, they were not used at all in the non-COVID group.

Ventilator-associated pneumonia that develops in intensive care patients causes an increase in morbidity and mortality by initiating the process leading to prolonged intensive care hospitalization, prolonged MV, sepsis, septic shock, and multi-organ failure. Similarly, the development of VAP in COVID-19 patients, which can cause all these clinical pictures, causes this picture to be more severe. With the high mortality and morbidity rates attributed to VAP, the implementation of any intervention that can reduce or prevent these infections will have significant benefits in terms of COVID-19 patients, healthcare capacity, and economic outcomes for hospitals. Among the current VAP prevention strategies, careful hand washing, raising the bed head, oral care using chlorhexidine, sedation breaks, subglottic drainage, and compliance with early removal from the ventilator will lead to better clinical results.^33^ In our study, we believe that the reasons for the higher rate of VAP in COVID-19 patients are the inability to keep the patient’s head raised (giving the patient a prone position), the need to continue sedation, and the inability to modify sedation due to severe respiratory failure in the patients.

## Conclusion

Ventilator-associated pneumonia development is more common in COVID-19 patients treated with MV than in non-COVID patients. The extra damage to be caused by VAP that develops on the already damaged lungs of the COVID-19 patients causes morbidity and mortality to be much higher. Since the clinical findings in these patients can be confused with the VAP findings, these findings are more difficult to diagnose. The predictive value of fever in the diagnosis is very low, and the production of the agent together with increased purulent sputum will be more valuable in terms of diagnosis. While diabetes mellitus is associated with higher mortality in VAP patients with COVID-19, we can say that other comorbidities have a similar effect on the development of VAP.

## Figures and Tables

**Table 1. t1-tjar-50-suppl1-s22:** Evaluation of Pneumonia Incidence Rates Among Groups

	COVID-19 VAP	Non-COVID VAP	
VAP	n (%)	n (%)	*P*
**Yes**	72 (52.2%)	54 (33.5%)	.001^*^
**No**	66 (47.8%)	107 (66.5%)	

Chi-square test, ^*^ *P* < .05.

**Table 2. t2-tjar-50-suppl1-s22:** Distribution of Operating Parameters

	Min-Max	Mean ± SD (median)
Fever (n = 123)	36.2-38.4	36.91 ± 0.52 (36.7)
**Leukocyte**	4190-48 800	15856.59 ± 7480.01 (14600)
Procalcitonin (n = 84)	0.04-56.6	8.85 ± 14.21 (2.5)
**Treatment duration (days)**	1-43	11.75 ± 5.7 (11)
Sedation duration (days) (n = 89)	1-31	10.98 ± 6.53 (10)
**MV duration (days)** (n = 122)	2-143	23.14 ± 23.53 (15)
**ICU duration (days)** (n = 125)	5-276	29.22 ± 31.98 (20)
	**n**	**%**
Body temperature (n = 123)		
**Normal**	114	92.7
**High**	9	7.3
**Leukocyte level**		
**Not normal**	104	82.5
**Normal**	22	17.5
**Procalcitonin level ** (n = 84)		
**Normal**	25	29.8
**High**	59	70.2
**Procalcitonin presence ** (n = 121)		
**No**	37	30.6
**Yes**	84	69.4
**Purulent sputum ** (n = 121)		
**No**	6	5
**Yes**	115	95
Sedation (n = 121)		
**No**	31	25.6
**Yes**	90	74.4
**ICU discharge type ** (n = 124)		
**Exitus**	97	76.9
**Hospitalized**	1	0.79
**Discharged**	28	22.2
Mortality (n = 124)		
**Survived**	29	23.1
**Exitus**	97	76.9

ICU, intensive care unit; MV, mechanical ventilation.

**Table 3. t3-tjar-50-suppl1-s22:** Evaluation of Study Parameters Between Cases with and without COVID-19

	Non-COVID Mean ± SD(median)	COVID-19 Mean ± SD (median)	*P*
**Treatment duration (days)**	13.48 ± 6.91 (12)	10.44 ± 4.2 (10)	**.010** **1** **,** *
**Sedation duration (days)**	10.9 ± 6.82 (10)	11.02 ± 6.43 (10)	**.836** **1**
**MV duration (days)**	34.53 ± 30.22 (23)	14.96 ± 11.93 (11)	**.000** **1,** *
**ICU duration (days)**	42.79 ± 42.93 (28)	19.22 ± 14.03 (14.5)	**.000** **1** *
	**n (%)**	**n (%)**	
**Fever level**			
**Normal**	47 (87)	67 (97.1)	**.037** **2,** *
**High**	7 (13)	2 (2.9)	
**Leukocyte level**			
**Not normal**	46 (85.2)	58 (80.6)	**.660** **3**
**Normal**	8 (14.8)	14 (19.4)	
**Procalcitonin level**			
**Normal**	14 (34.1)	11 (25.6)	**.536** **3**
**High**	27 (65.9)	32 (74.4)	
**Presence of procalcitonin**			
**No**	8 (16.3)	29 (40.3)	**.009** **3,** *
**Yes**	41 (83.7)	43 (59.7)	
**Purulent sputum**			
**No**	3 (5.6)	3 (4.5)	**.553** **2**
**Yes**	51 (94.4)	64 (95.5)	
**Presence of sedation**			
**No**	21 (40.4)	10 (14.5)	**.003** **3,** *
**Yes**	31 (59.6)	59 (85.5)	
**ICU discharge type**			
**Ex**	35 (64.9)	62 (86.1)	**-**
**Hospitalized**	1 (1.8)	0 (0)	
**Discharged**	18 (33.3)	10 (13.9)	
**Mortality**			
**Not ex**	19 (35.1)	10 (13.9)	**.012** **3,** *
**Ex**	35 (64.9)	62 (86.1)	

^1^Mann–Whitney *U* test.

^2^Fisher’s exact test.

^3^Continuity (Yates) fix.

^*^ *P *< .05.

ICU, intensive care unit; MV, mechanical ventilation.

**Table 4. t4-tjar-50-suppl1-s22:** Evaluation of Comorbidities Between COVID-19 and Non-COVID Patients

Comorbidities	Non-COVID n (%)	COVID-19 n (%)	*P*
**Comorbidity**	45 (83.3)	67 (93.1)	**.152**
**Congestive heart failure**	4 (7.4)	7 (9.7)	**.451** **2**
**Chronic obstructive pulmonary disease **	5 (9.3)	13 (18.1)	**.255** **3**
**Coronary artery disease**	11 (20.4)	15 (20.8)	**1.000** **3**
**Hypertension**	30 (55.5)	32 (44.4)	**.303** **1**
**DM**	14 (25.9)	17 (23.6)	**.929** **3**
**Dementia**	8 (14.8)	5 (6.9)	**.254** **3**
**Muscle disease**	0 (0)	2 (2.8)	**-**
**Bipolar disorder**	0 (0)	1 (1.4)	**-**
**Chronic renal insufficiency**	4 (7.4)	8 (11.1)	**.693** **3**
**Malignancy**	4 (7.4)	14 (19.4)	**.098** **3**
**AF**	4 (7.4)	3 (4.2)	**.343** **2**
**Parkinson’s**	2 (3.7)	5 (6.9)	**.355** **2**
**Cerebro vascular event**	9 (16.7)	5 (6.9)	**.152** **3**
**Asthma**	2 (3.7)	5 (6.9)	**.355** **2**
**Pulmonary embolism**	2 (3.7)	1 (1.4)	**-**
**Hyperlipidemia**	1 (1.9)	1 (1.4)	**-**
**Hypothyroidism**	0 (0)	2 (2.8)	**-**
**Epilepsy**	2 (3.7)	1 (1.4)	**-**
**Benign prostatic hypertrophy**	1 (1.9)	2 (2.8)	**-**
**Rheumatoid arthritis**	0 (0)	1 (1.4)	**-**
**Obesity**	0 (0)	1 (1.4)	**-**

^1^Chi-square test.

^2^Fisher’s exact test.

^3^Continuity (Yates) fix.

BPH, benign prostatic hypertrophy; CVE, cerebrovascular event; CRF, chronic renal insufficiency; HT, hypertension; CAD, coronary artery disease; COPD, chronic obstructive pulmonary disease; CHF, congestive heart failure.

**Table 5. t5-tjar-50-suppl1-s22:** Evaluation of Isolated Pathogens between Those with and without COVID-19

Isolated Pathogens	Non-COVID n (%)	COVID-19 n (%)	*P*
*Stereotrophomonas* *m* *altophilia*	7 (13)	9 (12.5)	**1.000** **1**
*Acinetobacter* *b* *aumannii*	35 (64.8)	40 (55.6)	**.387** **1**
*S* *taphylococcus* *a* *ureus*	4 (7.4)	5 (6.9)	**.592** **2**
*Pse* *u* *domonas * *a* *eruginosa*	11 (20.4)	10 (13.9)	**.469** **1**
*Escherichia* *c* *oli*	2 (3.7)	1 (1.4)	**-**
*Klebsiella* *p* *neumonia* *e*	11 (20.4)	13 (18.1)	**.922** **1**
*Raolitella* *o* *rnithindia*	0 (0)	1 (1.4)	**-**
*Enterobacter*	1 (1.9)	2 (2.8)	**-**
*Candida * *albicans*	0 (0)	2 (2.8)	**-**
**Gr+ diplococcus**	0 (0)	3 (4.2)	**-**
Gr− bacilli	1 (1.9)	0 (0)	**-**

^1^Continuity (Yates) fix.

^2^Fisher’s exact test.
